# Survival Analysis of Prefabricated Zirconia Crowns with and Without Pulpotomy in Primary Teeth: A Retrospective Cohort Study

**DOI:** 10.3390/children11111402

**Published:** 2024-11-19

**Authors:** Murad Alrashdi

**Affiliations:** Department of Orthodontic and Pediatric Dentistry, College of Dentistry, Qassim University, Buraydah 52571, Saudi Arabia; mu.alrashidi@qu.edu.sa

**Keywords:** prefabricated zirconia crown, pulpotomy, primary teeth

## Abstract

Background: Prefabricated Zirconia Crowns (PZCs) are increasingly preferred for restoring primary teeth due to their esthetic appeal and retention. However, their rigid, unmodifiable design requires precise tooth preparation, often leading to aggressive reduction and potential pulp exposure. Pulpotomy, a standard treatment for reversible pulpitis and mechanical pulp exposure, is sometimes employed before PZCs. While pulpotomy is not routinely performed, its use raises important considerations about the interplay between restorative procedures and pulp therapy in pediatric dentistry, particularly regarding the long-term restoration outcomes of PZCs. Purpose: This study aimed to investigate the impact of pulpotomy on the success rate of PZCs. Methods: We examined 81 anterior upper primary teeth treated with PZCs in children aged 2–5 years over a two-year period. Cases were divided into groups with and without pulpotomy. Follow-ups occurred at 6-month intervals, assessing clinical and radiographic outcomes. Analyses were performed using SPSS 25.0 software. The statistical significance was *p* < 0.05. Results: A total of 81 anterior primary teeth were included. Chi-square analysis showed no association between pulp therapy and PZC success (χ^2^ = 0.051, *p* = 0.822). The Kaplan–Meier survival analysis revealed comparable survival curves and the log-rank test showed no statistically significant difference in survival time between pulpotomy-treated and untreated groups (χ^2^ = 0.051, *p* = 0.821). Conclusions: Pulpotomy did not significantly affect the success rate of PZCs within 2 years.

## 1. Introduction

Prefabricated Zirconia Crowns (PZCs) are now increasingly preferred for the restoration of primary teeth owing to their superior qualities, including esthetic appeal, which satisfies the cosmetic needs of young patients [1]. Zirconia is typically classified based on the concentration of yttria, which influences its microstructure and mechanical properties. The most common types are 3Y-TZP, 4Y-PSZ, and 5Y-PSZ. Type 3Y-TZP offers the highest strength and toughness, making it ideal for posterior restorations where durability is essential [2]. Type 4Y-PSZ balances strength with improved translucency, making it suitable for both posterior and anterior applications [3]. Type 5Y-PSZ has the highest translucency but slightly reduced strength, making it preferred for highly esthetic anterior restorations [3]. Zirconia ceramics have high strength, fracture resistance, and biocompatibility [4,5]. Unlike other ceramics, zirconia exhibits a high flexural strength, with 3Y-TZP zirconia surpassing 900 MPa, making it highly resistant to fracture. In contrast, lithium disilicate, while known for its excellent esthetics and good strength, lacks the fracture resistance of zirconia. Similarly, feldspathic porcelain and leucite-reinforced ceramics, though offering high translucency and excellent esthetic outcomes, are more brittle and prone to chipping, especially in posterior load-bearing areas [4]. Zirconia’s combination of strength and biocompatibility makes it ideal for applications that require both durability and esthetics, especially in full-contour restorations where other ceramics might be susceptible to wear and fracture. Clinical indications for PZCs include extensive carious lesions, developmental defects, teeth with multiple surface involvement, and cases requiring full coverage restoration where esthetics is a primary concern [6]. However, one significant limitation of PZCs is the inability to modify these crowns once they are fabricated [7]. Unlike some other materials that allow for adjustments during the placement process, PZCs are manufactured by producers in predetermined sizes and shapes that cannot be altered, limiting the dentist’s ability to modify them chairside and necessitating careful selection and preparation of the tooth to fit the crown [7]. This requirement for accuracy often leads to a more aggressive tooth preparation process, increasing the risk of inadvertent pulp exposure during tooth preparation [7]. Although pulpotomy has been a standard treatment approach for primary teeth showing signs of reversible pulpitis, where the infection or inflammation is limited to the coronal part of the pulp, some dentists contend that pulpotomy is necessary before PZCs to address inadvertent pulp exposure that may occur during tooth preparation. Exposed pulps have been successfully treated with pulpotomy with the assumption that the inflammation caused by the carious or mechanical exposure is only limited to the coronal pulp [8,9]. This has been a predominant treatment approach in primary teeth with a high success rate [10]. The intersection of PZC restorations and pulp therapy in primary teeth presents an intriguing area of study in pediatric dentistry. While pulpotomy is not routinely performed prior to PZC placement, its application in cases of pulp exposure or pulpitis may have significant implications for the long-term success of the restoration. This raises several important questions about the outcomes of PZC restorations, particularly when comparing teeth that have undergone pulpotomy with those that have not required pulp therapy. These questions highlight the complex interplay between restorative procedures and pulp management regarding whether the performance of pulpotomy in the indicated cases enhances the longevity and success rate of PZC restorations, and if there are differences in complication rates between pulpotomy-treated and untreated groups. Therefore, the primary objective of this study is to investigate the impact of pulpotomy on PZC outcomes by comparing teeth that underwent pulpotomy due to pulpitis with those that did not require pulpotomy prior to PZCs. By examining the outcomes of pulpotomy and PZC restorations in these two groups, we aim to provide evidence to guide treatment decisions and optimize the restoration of primary teeth with PZCs.

## 2. Materials and Methods

### 2.1. Study Design

This study employed a retrospective cohort design utilizing pediatric dental records of children who attended a dental clinic at Qassim University spanning two years from 2021 to 2023.

### 2.2. Participants

This retrospective cohort study analyzed data from 90 primary upper anterior teeth treated with Prefabricated Zirconia Crowns (PZCs) over a two-year period. The teeth were from 90 pediatric patients aged between 2 and 5 years, with at least one treated patient. The study focused exclusively on primary upper anterior teeth (both central and lateral incisors).

### 2.3. Sample Selection

The sample was selected based on the availability of eligible cases from the dental records during the two-year study period. Convenience sampling was used, where all qualifying patients treated within the study timeframe were considered for inclusion. From the 90 initial cases, which constitute the entire cohort of patients treated with PZCs at our clinic, the final sample size was reduced to 81 anterior primary teeth after applying the inclusion and exclusion criteria. Of the nine (9) cases excluded from the final analysis, four had incomplete clinical and radiographic records that prevented proper outcome assessment. Three crowns were performed on primary molars, and two (2) crowns were lost due to traumatic injury shortly after placement. The selection process for zirconia crown restorations at our facility primarily considered the tooth’s clinical presentation and pulp vitality. Teeth with normal pulp or reversible pulpitis qualified for zirconia crowns. Pulpotomy was only performed if pulp exposure occurred during preparation or due to caries. For teeth without pulpotomy, thorough clinical and radiographic evaluations are conducted to rule out deep caries or pulp involvement before crown placement. To mitigate risks of undetected minor pulp exposures at horn tips, we pay close attention to the extent of tooth reduction needed for zirconia crown fit. We employ conservative preparation methods to preserve tooth structure and minimize the chance of accidental pulp exposure in non-pulpotomized teeth.

### 2.4. Inclusion and Exclusion Criteria

The following were applied to select appropriate cases for inclusion in the study:

Inclusion Criteria:

1. Only teeth treated with zirconia crowns within the specified two-year study period were included;
2. Teeth that had not been previously restored with other types of crowns were included;
3. Teeth with normal pulp or reversible pulpitis were included;
4. Cases with complete and well-documented clinical and/or radiographic data were included.

Exclusion Criteria:

1. Zirconia crowns that fell off during initial cementation or due to trauma were excluded;
2. Crowns that exhibited poor fit or other quality issues during initial cementation were excluded;
3. Primary molars were not included in this study.

### 2.5. Treatment Procedures

Pulpotomy therapy in this study was performed on teeth exhibiting signs of reversible pulpitis. After caries removal, the coronal pulp was carefully extirpated using sterile instruments, followed by the application of Mineral Trioxide Aggregate to the radicular pulp stumps. Hemostasis was achieved using sterile cotton pellets, and the pulp chamber was sealed with a layer of reinforced zinc oxide eugenol. After preparation, the tooth was cleaned, dried, and a self-adhesive resin cement was used to cement the crown. The crown was filled with cement, seated on the prepared tooth, and held under pressure until the initial set. Excess cement was removed, and the occlusion was adjusted as necessary. All restorations were performed using NuSmile^®^ (Houston, TX, USA) zirconia crowns. All procedures were performed by one dentist.

### 2.6. Data Collection

**Figure 1:**
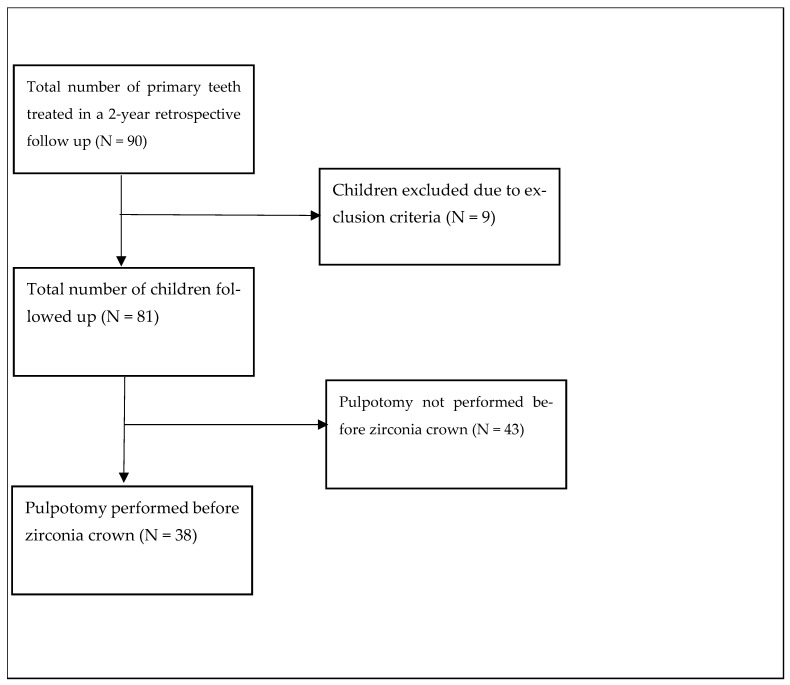
Study design.

Data were extracted from electronic dental records, encompassing patient demographics (age and gender), details of PZCs and pulpotomy before PZCs, and initial pulp condition. The pulp condition was classified as normal pulp, reversible pulpitis, symptomatic or asymptomatic irreversible pulpitis, or necrotic pulp based on a combination of clinical examination, patient-reported symptoms, radiographic evaluation, and pulp vitality tests where applicable. Normal pulp was defined as absence of pain or discomfort and radiographs showed no periapical radiolucency with an intact lamina dura. Reversible pulpitis was defined as sharp but quickly subsiding pain to stimuli such as cold, with normal radiographs except for caries. Symptomatic irreversible pulpitis was characterized by spontaneous, lingering, often severe and throbbing pain, with radiographs showing deep caries with normal periapical area. Asymptomatic irreversible pulpitis showed positive responses to sensibility tests without significant discomfort, and radiographs indicated deep caries without periapical radiolucency. Necrotic pulp exhibited no response to sensibility tests, and radiographs revealed periapical radiolucency, disrupted lamina dura, and widened periodontal ligament space. These diagnoses were made and documented by the treating dentists at the time of initial examination. To ensure consistency, only cases with clear documentation of initial pulp condition were included in the analysis. Follow-up information was collected over a 2-year period, with patients recalled at 6-month intervals. Success or failure outcomes were explicitly documented based on predefined criteria. These included clinical signs and symptoms such as pain, gingival abscess, fistula openings, radiological findings, and abnormal mobility. Importantly, the absence of documented failure was not automatically interpreted as success. Instead, active assessment and documentation of both successful and failed cases were required at each follow-up visit (Figure 1).

### 2.7. Primary Outcome Measures

For primary teeth that underwent pulpotomy, success criteria included absence of clinical symptoms and radiographic evidence of healthy periapical and furcal areas. Clinical examinations assessed for pain, gingival abscess, and fistula openings. Radiographic evaluations, including periapical and bitewing radiographs, were analyzed at baseline and each follow-up to assess periradicular health, restoration integrity, and potential complications such as periapical or furcal radiolucency, root resorption, and changes in lamina dura or periodontal ligament space. PZC restoration success was evaluated based on retention, marginal integrity, and absence of secondary caries. Outcomes were analyzed separately for teeth with and without pulpotomy, with success rates calculated based on combined clinical and radiographic findings.

### 2.8. Statistical Analysis

Descriptive statistics including frequencies, percentages, means and standard deviations were obtained. An independent *t*-test was employed to compare differences in age between pulpotomy-treated and untreated groups. The chi-square test was utilized to assess the association between pulpotomy and the success of PZCs. Binary logistic regression analysis was thereafter conducted to examine the association between pulpotomy and the success of PZCs with age, gender, and initial pulp condition as covariates. Adjusted odds ratio and 95% confidence intervals were calculated. The Kaplan–Meier survival analysis was performed to estimate the probability of survival of zirconia crowns over time, with log-rank tests used to compare survival curves between pulpotomy-treated and untreated groups. The null hypothesis was that there is no statistically significant difference in success rates of zirconia crown between primary teeth that underwent pulpotomy and those that did not before restoration. The data were analyzed using SPSS 25.0 software (IBM Corp., Armonk, NY, USA)

### 2.9. Ethical Considerations

This study was conducted by ethical guidelines and the approval for the study was obtained from The Institutional Review Board (IRB) of Qassim University, protocol number 22-4-13. Patient confidentiality was maintained throughout the data collection and analysis process.

## 3. Results

**Figure 2:**
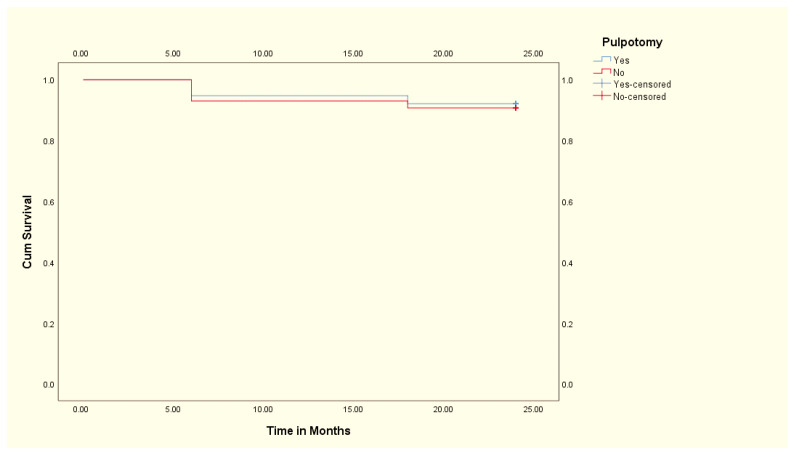
Kaplan–Meier survival curve showing the comparison between PZC with and without pulpotomy.

In this study, a total of 81 primary teeth were included from 81 participants, with males comprising 56.8% (46 out of 81). Among these, 46.9% (38 out of 81) underwent pulpotomy before PZCs. All primary teeth that underwent pulpotomy presented with reversible pulpitis and exhibited initial pulp conditions indicative of inflammation or infection. Overall, at the 24-month follow-up, it was observed that 74 out of the initial 81 PZCs placed had survived without abnormal mobility, pain, gingival abscess, or fistula opening, indicating a success rate of approximately 91.4%. In this study, 7 out of the 81 zirconia crowns failed over the two-year follow-up period. The primary causes of failure included debonding, which accounted for the majority of cases (4). Secondary caries and subsequent pain and swelling were observed in two (2) cases. Gingival abscesses were noted in one (1) case. The Chi-square test revealed no statistically significant association between pulpotomy and the success of PZCs within the 24-month period (χ^2^ = 0.051, *p* = 0.822). Subsequently, the results of our binary logistic regression found that age (OR: 0.644, 95% CI: 0.291–1.423, *p* = 0.277), gender (OR: 0.603, 95% CI: 0.123–2.954, *p* = 0.533), and initial pulp condition (OR: 1.138, 95% CI: 0.233–5.533, *p* = 0.873) were not independent predictors of the success of PZCs. There was no statistically significant difference in age between pulpotomy-treated and untreated groups [t (79) = 0.625, *p* = 0.534]. These results suggest that, within the parameters of the study, neither age, gender, initial pulp condition, nor the performance of pulpotomy independently influenced the likelihood of the success of PZCs. Lastly, the survival functions for the PZCs with pulpotomy and those without pulpotomy were plotted using Kaplan–Meier (Figure 2). The mean survival time of the PZCs with pulpotomy (22.9 ± 0.7 months) was slightly higher than the mean survival time of the PZCs without pulpotomy (22.6 ± 0.7 months). The log-rank test, assessing the significance of the differences between the survival curves, yielded a non-significant result (χ^2^ = 0.051, *p* = 0.821), indicating no statistically significant difference in PZC survival between the pulpotomy-treated and untreated groups.

## 4. Discussion

Recent meta-analyses and systematic reviews have reinforced the positive results associated with zirconia crowns. These studies have evaluated various aspects of PZC performance, including their impact on periodontal health, structural integrity at the crown margins, color stability, and esthetics [11,12,13]. In this study, we investigated the survival analysis of PZCs, with a particular focus on the impact of pulpotomy on crown outcomes. Our findings provide valuable insights into the effectiveness of these treatment approaches in pediatric dentistry. Overall, we observed a high success rate of PZCs, a finding similar to a systematic review by Pjetursson and colleagues [14]. Our analysis also revealed no statistically significant difference in the success rates of PZCs between teeth that underwent pulpotomy and those which did not. This suggests that the performance of pulpotomy prior to PZC placement may not influence the long-term outcomes of PZCs. However, the limited literature has examined the impact of pulp treatment on the survival rate of PZCs. In a study by Gamze Topçuoğlu et al., the survival rate of zirconia crowns in primary anterior teeth appeared to be high, with no significant difference in survival between pulp-treated and untreated teeth [15]. Similarly, a retrospective evaluation of zirconia crowns in children treated under general anesthesia found that crowns placed on teeth after pulp therapy were more likely to fail than those placed on teeth without pulp therapy, although the overall success rate was high [16]. The findings from our analysis provide evidence against pulpotomy as a significant determinant of the long-term success of PZCs. Some dentists have suggested that because of aggressive tooth preparation required for PZCs, pulpotomy is necessary due to the high risk of mechanical pulp exposure. Despite the theoretical rationale behind this approach as a proactive measure to preserve pulp vitality and enhance the success of dental restorations, the results of this study suggest otherwise. While it may initially seem necessary to perform pulp treatment on teeth indicated for zirconia crowns, there is no definitive rationale for this approach when dental caries do not reach the pulp [17]. Clinical examination often serves as the primary means to accurately identify the pulp status of primary teeth. In this study, and our clinical practice, the decision to perform pulp therapy was based solely on whether clinical pulp exposure was observed.

The relationship between pulp therapy and crown survival remains a subject of ongoing investigation. Several factors may influence the survival rates of restorations following pulp treatment, including the type of pulp therapy performed, the remaining tooth structure, and the quality of the final restoration. Research suggested that the success of PZCs on pulp-treated teeth may be influenced by the timing of crown placement relative to pulp therapy completion, with better outcomes observed when crown placement is delayed to ensure adequate healing [18]. A recent systematic review and meta-analysis demonstrated that, while both pulpotomy and pulpectomy are viable treatment options for primary teeth, pulpotomy showed more favorable outcomes in terms of clinical and radiographic success rates [19]. This finding is particularly relevant when considering crown placement, as the preservation of the radicular pulp in pulpotomy cases may contribute to maintained tooth vitality and potentially better long-term crown stability. The study also highlighted that pulpotomy procedures are generally less technique-sensitive and time-consuming compared to pulpectomy, which could influence the overall treatment planning when considering PZC placement. The lack of significant differences in survival time between pulpotomy treated and untreated crowns in our study implies that factors beyond pulpal intervention may have played a more substantial role in the durability and success of zirconia crowns. For example, a study revealed that the type of cement used and patient-related factors might have a minimal influence on the survival and success rates of zirconia crowns [20]. Furthermore, the potential impact of different obturation materials and techniques on crown adhesion warrants further investigation.

Over the two-year retrospective follow-up period, a small number of zirconia crowns failed. The most common cause of failure was debonding, despite careful crown preparation, highlighting that cement remains the principal source of retention for these restorations. Secondary caries and abscess formation were uncommon complications, though their occurrence emphasizes the importance of proper crown fit and cementation technique. Despite these failures, the projected success rate for zirconia crowns in this study was high, exceeding that of composite resin-based strip crowns and showing comparable, if not superior, performance to stainless steel crowns when debonding issues are not considered [21,22,23]. The low incidence of biological complications, such as secondary caries and abscess formation, supports the biocompatibility of zirconia as a restorative material for primary teeth. This observation is particularly significant given the challenges of maintaining optimal oral hygiene in pediatric patients. The smooth surface characteristics of zirconia may contribute to reduced plaque accumulation compared to other restorative materials, potentially explaining the low rate of biological complications observed [5].

Taken together, our findings call into question the role of pulpotomy in improving the outcomes of PZCs. We highlight the need for more understanding of the multifactorial nature of dental restoration outcomes, with consideration given to other variables that may influence the longevity of PZCs. Therefore, while pulpotomy may have its benefits, the evidence presented in this study suggests that its impact on the success of PZCs may be less significant. Additionally, pulpotomy may offset the esthetic benefits of PZCs as revealed by one study that teeth that underwent pulpotomy exhibited inferior color matching with neighboring teeth compared to teeth that did not undergo pulpotomy [24]. Dentists should carefully weigh the potential benefits and risks of pulpotomy when considering treatment options for patients requiring crown restoration. While our research corroborates the impressive success rates of zirconia crowns reported in the existing literature, it is important to acknowledge certain practical limitations that may restrict their widespread adoption in everyday dental practice. These constraints include the need for meticulous tooth preparation, the higher financial investment required, and the technique-sensitive nature of PZC placement. These factors collectively present challenges that might hinder the routine use of PZCs, despite their clinical efficacy.

The limitations of this study include the retrospective design, which may introduce selection bias. The relatively small sample size and short follow-up period may also limit the generalizability of the findings. Another significant limitation of this study is the inability to account for several potentially confounding variables that could influence the success of zirconia crown restorations. While we considered age, gender, and initial pulp condition in our analysis, other crucial factors such as oral hygiene practices, parental motivation for dental care, and dietary habits were not systematically recorded or analyzed in this retrospective study. These factors could play a significant role in the long-term success of crown restorations. For instance, poor oral hygiene could lead to secondary caries or gingival inflammation around the crown margins, potentially compromising the restoration’s integrity [25]. Similarly, high sugar consumption could increase the risk of recurrent decay [23]. Parental motivation might affect adherence to post-treatment care instructions, which could impact the longevity of the restorations. The absence of data on these variables limits our ability to fully explain the factors contributing to crown success or failure. Larger-scale prospective studies with longer follow-up periods which includes electively pulpotomized teeth are also warranted to determine whether pulp therapy is necessary for normal pulps before PZCs, a finding that we were unable to examine based on our data.

## 5. Conclusions

This retrospective cohort study demonstrated a high overall success rate of PZCs over two years, with no significant differences between teeth that underwent pulpotomy and those that did not. Neither pulpotomy nor factors such as age, gender, and initial pulp condition independently predicted PZC success. While pulpotomy remains valuable for managing mechanical pulp exposure and pulpitis, our findings suggest that its routine application before PZC placement may not be necessary solely to enhance crown longevity. Despite the study’s limitations, including its retrospective nature and relatively short follow-up period, these results indicate that the decision to perform pulpotomy should be guided by specific clinical indications rather than as a routine preparatory step for PZC restoration.

## Data Availability

The data supporting the findings of this study are available from the corresponding author upon reasonable request. Due to privacy concerns, individual patient data cannot be made publicly available.
